# Supplementary figures and images for: Thermal traits for reproduction and recruitment differ between Arctic and Atlantic kelp Laminaria digitata
Source: PLoS One. 2020 Jun 30;15(6):e0235388. doi: 10.1371/journal.pone.0235388 (PMC7326501; doi:10.1371/journal.pone.0235388)

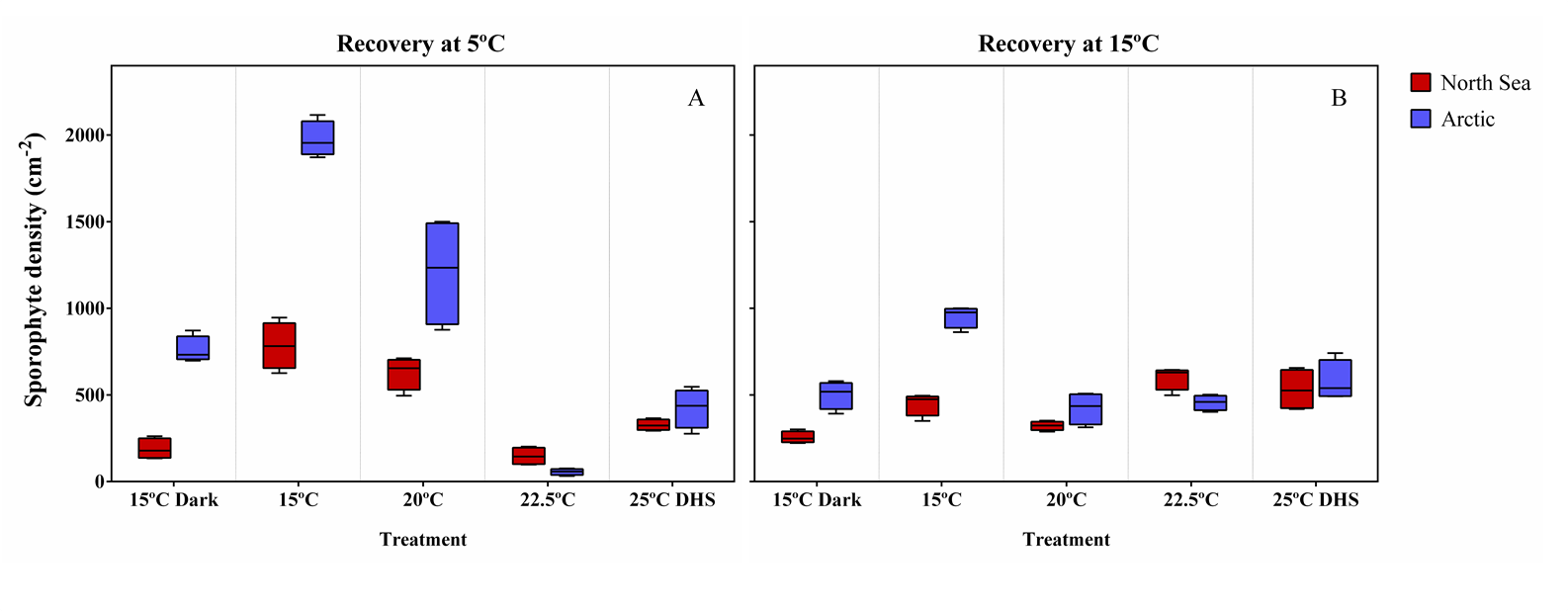

Supplement: S1 Fig — Absolute number of sporophytes from the North Sea and Arctic populations of Laminaria digitata after 27 days of recovery at 5°C (A) and 15°C (B) from different treatments (15°C dark, 15°C, 20°C, 22.5°C and 25°C DHS). Box plots with median, boxes for 25th and 75th percentiles and whiskers indicating min and max values (n = 4). (TIF) [file pone.0235388.s001.tif]

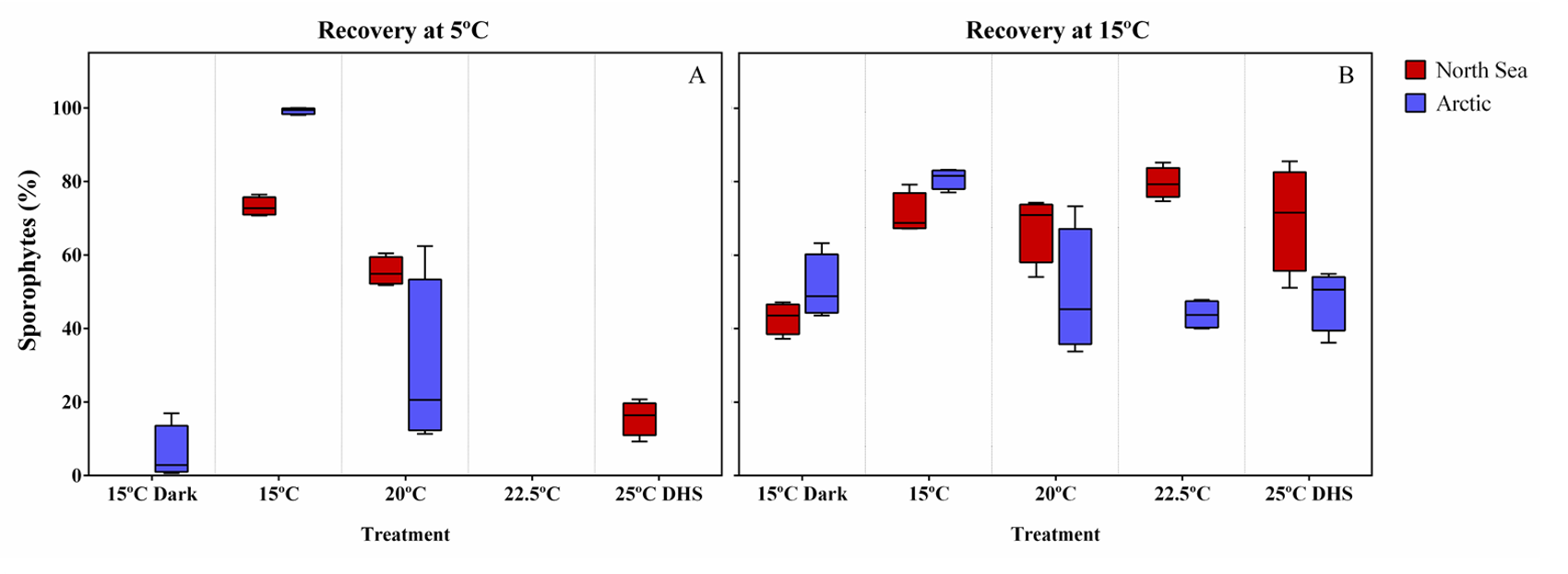

Supplement: S2 Fig — Percentage of female multicellular gametophytes with juvenile sporophytes from the North Sea and Arctic populations of Laminaria digitata after 20 days of recovery at 5°C (A) and 15°C (B) from different treatments (15°C dark, 15°C, 20°C, 22.5°C and 25°C DHS). Box plots with median, boxes for 25th and 75th percentiles and whiskers indicating min and max values (n = 4). (TIF) [file pone.0235388.s002.tif]
