# Supplementary material for: Thermal traits for reproduction and recruitment differ between Arctic and Atlantic kelp Laminaria digitata
Source: PLoS One. 2020 Jun 30;15(6):e0235388. doi: 10.1371/journal.pone.0235388 (PMC7326501; doi:10.1371/journal.pone.0235388)
Supplement: Attachment — Submitted filename: Response to Reviewers.docx [file pone.0235388.s004.docx]

**I have two reviews on my desk now, but unfortunately with different recommendations: accepted with minor comments (newly invited reviewer3), and a major revision with more serious remarks (reviever2); some of which I will bring up below:**

**1) Statistical analyses were not performed rigorously. In this case, I reviewed the statistical analyses by myself and the comments could be found below in the 'Editorial Statistical revision' section.**

We very much appreciate the Editor’s suggestions with regard to the statistical analyses. All the recommended changes have now been implemented to improve the statistical analyses.

**2) The results do not support the conclusions. The stress levels were confused by the authors with temperature levels. Studied populations were not directly comparable because there were different stress levels on each (the Arctic 10-20°C increase vs Helgoland 2-7°C increase). Therefore, the conclusion about a higher stress tolerance in Helgoland population is not supported and could be the result of different experimental design.**

This point is addressed directly in our responses to reviewer #2

**3) The experimental design and results did not allow to conclude about evolutionary consequences related to adaptation, as the authors suggested. The results should be interpreted more directly and as straight as possible. Moreover, the response for acclimation/acclimatisation should not be confused with adaptation.**

This point will be addressed directly in our responses to reviewer #2.

Editorial Statistical revision

**1) This study was performed on the insufficient number of replications. It is a statistically inappropriate idea to have more factor levels than samples per level (n=4). Such sample size is also insufficient to trust S-W normality test and, what is more important, it reduces the statistical power of ANOVA (likelihood of rejecting the H0 when it actually should be rejected, a type II error).**

We accept the criticism regarding the insufficient number of replications used in this study and therefore we implemented all the changes recommended by the Editor to improve the statistical power of the analyses (please see the answers below).

**2) For the reason of heterogeneity of variance in factor levels and both types of distribution skewness in the data, the Box-Cox transformation should be performed prior to all analysis.**

Only the normalized sporophyte density showed heterogeneity of variance and distribution skewness and therefore these data were transformed via Box-Cox as suggested.

**3) Because of low number of replications, PERMANOVA with PERMPDISP should be applied instead of all ANOVA type analyses, which would also help to unify the interpretation of the results. If the PERMDISP reviled heterogeneity, a short information (*) in the tables is required and short comments in results or discussion about how data quality could influence the results.**

We appreciate the Editor’s suggestion and all data were analysed with PERMANOVA and PERMDISP to unify the results. Whenever PERMDISP revealed heterogeneity in significant main effects or interactions found with PERMANOVA, this information was included in the manuscript.

**4) Despite the first reviewer’s suggestion, my recommendation is to use the same level of alpha α = 0.05 in all models/tests to unify their sensitivity, and to allow for comparisons between the models and results interpretation. The significance level should be mentioned only once in the methods section and removed from the results and tables.**

The Editor’s suggestion was accepted. The level of alpha α = 0.05 was implemented in all tests. This information was included only in the methods section (Line 312).

**5) In all PERMANOVA tables the SS for residuals (unexplained variation) should also be shown. It allows the readers to calculate the percent of total variation explained by tested factors if needed.**

As suggested, the values of SS for residuals were included in all PERMANOVA tables.

**6) In all tables the exact value of probability should be presented with the exception of p<0.001. The values of SS, MS and pseudo-F should be rounded to two decimal places.**

As recommended, the exact value of probability was shown in the tables and the SS, MS and pseudo-F values were decreased to two decimal places.

**7) The “Statistical analysis for” phrase should be removed from all tables’ captions and the name of analysis - PERMANOVA should be added. Also “Post-hoc analyses were performed using pair-wise t-test comparisons” should be changed to “The post-hoc results are presented in Fig. X’.**

The suggestion of the Editor was accepted and we have replaced “statistical analysis” by “PERMANOVA” and “Post-hoc analyses were performed using pair-wise t-test comparisons” by “The post-hoc results are presented in Fig. X” in all tables.

**Reviewer #2:**

**The authors downplayed the experimental concerns that the common garden experiment exposed the Arctic population to higher temperature stress (10-20°C increase) compared to the Helgolandic population (2-7°C increase), which has an implication on all response variables measured and consequently on the interpretation of results, (indirectly) suggesting that Helgoland population is more tolerant and Arctic population more susceptible to ocean warming.**

The comments of the reviewer refer to a different research question than the one in this paper. This paper aims to discover if different populations of the same species have distinct thermal tolerance limits and responses. This paper never did aim to test local responses to future effects of global warming (by comparing increases in temperatures of the same number of degrees above the temperature in which populations live locally). The referee’s comments are very interesting for any other paper with a research question about effects of oceanic warming among distinct populations, but are not relevant to this paper which asks the research question of whether individuals of the same species have distinct thermal tolerance range limits, as hypothesized if there is local selection in populations living in distinct habitats.

**The authors rebutted that “This paper does not aim to investigate whether populations will survive x degrees above their summer temperature. It aims to investigate whether this species has distinct thermal tolerances depending on the population of that same species; i.e., whether distinct populations of the same species have any functional differences at any temperatures. It is not an ecological question, it is an evolutionary question.”**

**The fact is they are comparing the two populations. How could they suggest one population is better than the other in tolerating stressful temperature condition, when absolute temperature values were used i.e. biased towards the habit temperature of one population and disregarded the ambient maximum summer temperatures the different populations are exposed to? The experiment could have designed +2, +4, +8, +16 °C increase relative to their highest summer temperature.
Organismal and/or population responses to ecological stress factors have evolutionary consequences. It seems inappropriate to suggest this study is answering evolutionary question but not ecological question. How does the experimental design answer which evolutionary question? The above contention needs to be contextualize in the paper.**

**The authors are advised to reassess or tone down their data interpretation and discussion relative to the limitation of their experimental design.**

By comparing populations near the cold and warm edges of the thermal envelope for this species, the goal is rather to test whether phenotypic traits in response to a common thermal stressor are stable over the species range, or whether they differ between populations from distinct environments, suggesting an underlying genetic component, that is an adaptive change between populations. In order to do this, a common garden approach was taken to remove as far as possible the effects of environmental history, hardening or pre-acclimation, so that they were exposed to the experimental conditions after living in a common thermal habitat for 3 years in the exact same temperatures. This is a standard approach to detect adaptation rather than acclimation. A 3-years common garden culture of haploid meiotic products (i.e., gametophytes) derived from the collected material, means that the cells under the experimental treatments had been dividing and growing in the exact same temperatures despite coming from distinct populations, as necessary to distinguish adaptation from acclimation. This is the best design for the stated null hypothesis that thermal phenotype is stable across the species thermal envelope, versus the alternative hypothesis that it differs between populations and that difference remains even when they have been living in the exact same thermal conditions. This design, by exposing the experimental material to a long-term common acclimation period followed by a common experimental design, therefore allows the inference of a genetic basis, that is adaptation and not acclimation, linked to past evolutionary history and selective forces, acting on the source population.

The reviewer suggests a design for an entirely different research question than ours outlined above, namely to assess whether temperature changes of a common delta magnitude relative to those prevailing at the population site would have a similar response magnitude and thereby similar or different ecological effects on each population. Although this is a meaningful research question, we did not follow this idea as we already possess a series of published and unpublished pre-information (Bolton and Lüning 1982, tom Dieck 1992, 1993, Bartsch et al. 2013, Martins et al. 2017, Franke 2019, King et al. 2019, Liesner et al. under revision) that suggest subtle response differences between populations of *Laminaria digitata* with respect to their thermal performance. Thus, the proposed delta treatments possibly would have generated quite foreseeable responses with the Helgoland individuals losing their fertility at sub-lethal temperatures and dying when surpassing their lethal limits and the Spitzbergen individuals entering their near optimal conditions. With our design and research question, in contrast, we are able to better resolve one aspect of the general response width of the meta-population of the investigated species which are e.g. required by modellers (see remark rev 3). We added a few words to the introduction (Line 91) highlighting the differences between these two research questions, ours more from a mechanistic point of view and the delta treatments concept better showing the direct ecological impacts under global warming, which are both relevant. We thereby hope to better inform the reader how to discriminate these two research cases that may not have been clear enough in our last manuscript version.

Finally, an interesting remark is that such experiments have both evolutionary implications (with regard to functional diversity within the species) and ecological implications. This is because unique thermal phenotypes or variants near the warm edge, if lost due to future climate impacts, either from extreme events or general poleward range shifts, may not be easily replaceable by natural migration or management actions from more northern populations.

Bartsch I, Vogt J, Pehlke C, Hanelt D. Prevailing sea surface temperatures inhibit summer reproduction of the kelp *Laminaria digitata* at Helgoland (North Sea). J Phycol. 2013; 49: 1061-1073.

Bolton J, Lüning K. Optimal growth and maximal survival temperature of Atlantic *Laminaria* species (Phaeophyta) in culture. Mar Biol. 1982; 66: 89-94.

Franke, K. (2019). Performance of different life cycle stages of the Arctic *kelps Laminaria digitata* and *Saccharina nigripes* along temperature gradients. Master's thesis. Bremen, Germany: University of Bremen.

King NG, McKeown NJ, Smale DA, Wilcockson DC, Hoelters L, Groves EA, et al. Evidence for different thermal ecotypes in range centre and trailing edge kelp populations. J Exp Mar Bio Ecol. 2019; 514-515: 10-17.

Liesner D, Fouqueau L, Valero M, Roleda MY, Pearson GA, Bischof K, Valentin K, Bartsch I. Heat stress responses and population genetics of the kelp *Laminaria digitata* (Phaeophyceae) across latitudes reveal differentiation among North Atlantic populations. Submitted

Martins N, Tanttu H, Pearson GA, Serrão EA, Bartsch I. Interactions of daylength, temperature and nutrients affect thresholds for life stage transitions in the kelp *Laminaria digitata* (Phaeophyceae). Bot Mar. 2017; 60: 109-121.

tom Dieck I. North Pacific and North Atlantic digitate *Laminaria* species (Phaeophyta): hybridization experiments and temperature responses. Phycologia. 1992; 31: 147-163.

tom Dieck I, de Oliveira EC. The section Digitatae of the genus *Laminaria* (Phaeophyta) in the northern and southern Atlantic: crossing experiments and temperature responses. Mar Biol. 1993; 115: 151-160.

**Reviewer #3:**

**This study examines the effects of different warming treatments on survival and performance of early life stages of kelp species from northern and southern range edges. The work is comprehensive and the results highly interesting, especially the larger conclusion that there are differences in thermal tolerances of geographically different populations of a species. Modeller often use a single thermal tolerance to predict range expansions or retractions of species with climate change, and results such as these challenge these simplisitic approaches and are highly important. The authors have done a good job of addressing the reviewers’ comments. However, I suggest that the temperature treatments are heat spikes, which is consistent with the Hobday et al. definition of extreme temperature events shorter than 5 days. I would also mention heatwaves in the ms again (e.g. revert to previous wording on line 62). The removal of this from the manuscript is a missed opportunity to tie these experiments in with the larger literature on MHWs.**

Because our research question is only and simply about discovering whether populations acclimated to common thermal conditions still remain different in thermal responses, at a particular set of temperatures in which such differences could be expected to happen, it is not really important for the question whether the tested set is called a heat wave or not. We are happy to leave the description of the experimental temperatures as it is, or to call it a heat wave. It really does not affect the research question or the conclusions, so we leave it up to the editor to decide whether to use this designation or not, given the disagreement among reviewers.

Minor comments by line.

- **Line 24. Arctic.**

As recommended, Artic was corrected to Arctic.

- **Line 128 5 sporophytes is not that many. Can you justify why so few adults are representative of the larger populations in these areas?**

We would like to clarify that we did not use sporophytes but rather male and female gametophytic progeny of each sporophyte. We agree that it would be preferable to use a wider gene pool from more parental sporophytes for the comparison of populations, but establishment of a next generation of individual gametophyte cultures is very labour- and time-consuming and this was the maximum manageable number of replicates. To generate sufficient vegetative gametophyte material to perform a replicated experiment such as ours requires at least 1-1.5 years (Bartsch 2018). In addition, we need the cultivation space. Thus to isolate and culture a sub-set of individual isolates from 5 sporophytes represents a good cost-time-space balance. The advantage of using individual gametophyte cultures is to investigate isolates from different populations/sites at the same time and cultivated under common conditions since the meiospore stage, thereby allowing to distinguish adaptation from acclimation/environmental history.

- **Line 266. didn’t should not be contracted here and throughout.**

In accordance with the reviewer comment, didn’t was changed to did not throughout the manuscript.

- **Table 1. report error**

As suggested, the values for residuals or errors were included in all statistical tables.

- **Line 382. For clarity, be more specific as to how they influence recruitment capacity.**
  As recommended, this information was included in the text (Line 439).
- **Line 385. Similar to what? Would be helpful to be more specific.**

Similar recruitment patterns were observed in the normalized sporophyte density (Fig. 5) and the relative presence of female multicellular gametophytes with sporophytes (S2 Fig). As suggested, this information was included in the manuscript (Line 444).

- **Table 4. Check significant digits, there is no need for 3 decimal places.**

As recommended, the decimal digits were decreased in all statistical tables.

- **Line 448. 28 °C conditions are survived for…**

The gametophytes of *L. digitata* survived at 28ºC for 1 day as already described in the text. However, we recognize that this sentence was not clear and, therefore, we clarified this in the discussion section (Line 516).

- **Line 452. Not sure you need (=L. schinzii) here, the species name pallida is established no?**

Indeed, the species name *L. pallida* is established; however the study that we cited in the text was performed by tom Dieck in 1993, when it was still known as *L. schinzii*. Therefore, we decided to keep both the current and previous species names, so that the readers that are not familiar with the species name change can associate the tom Dieck (1993) work by realizing that *L. schinzii* was in fact *L. pallida*.

- **Line 470. Why are the species names reported in this way… ‘L. schinzii (=L. pallida) [30]; Eckloniopsis’ ? This is not consistent with previous paragraph.**

We agree with the Reviewer comment and we reported the current and previous species names in a consistent way thorough the manuscript, i.e., current species name [formerly known as]. *L. pallida* [=*L. schinzii*)] and *Ecklonia radicosa* [=*Eckloniopsis radicosa*] (Line 538).
