# Supplementary material for: Thermal traits for reproduction and recruitment differ between Arctic and Atlantic kelp Laminaria digitata
Source: PLoS One. 2020 Jun 30;15(6):e0235388. doi: 10.1371/journal.pone.0235388 (PMC7326501; doi:10.1371/journal.pone.0235388)
Supplement: Attachment — Submitted filename: Response to Reviewers.docx [file pone.0235388.s005.docx]

**Answers to the Academic Editor and Reviewer comments:**

**Academic Editor:**

**One final minor remark, please adjust the length of the abstract to the requirements of the journal. Which allow me to finally accept the manuscript.**

**Reviewer 2:**

**Authors adequately addressed the concerns and supplied information in the introduction. Abstract is too long. Please edit concisely.**

As recommended, the abstract was shortened to less than 300 words to meet the publication requirements of *PLOS ONE*.
